# *Trypanosoma vivax* GM6 Antigen: A Candidate Antigen for Diagnosis of African Animal Trypanosomosis in Cattle

**DOI:** 10.1371/journal.pone.0078565

**Published:** 2013-10-25

**Authors:** Davita Pillay, Julien Izotte, Regassa Fikru, Philipe Büscher, Hermogenes Mucache, Luis Neves, Alain Boulangé, Momar Talla Seck, Jérémy Bouyer, Grant B. Napier, Cyrille Chevtzoff, Virginie Coustou, Théo Baltz

**Affiliations:** 1 Microbiologie fondamentale et Pathogénicité, UMR 5234, Centre National de la Recherche Scientifique (CNRS), Université Bordeaux Segalen, Bordeaux, France; 2 College of Veterinary Medicine, Addis Ababa University, Debre Zeit, Ethiopia; 3 Department of Biomedical Sciences, Institute of Tropical Medicine, Antwerp, Belgium; 4 Faculty of Bioscience Engineering, Department Biosystems, KU Leuven, Leuven, Belgium; 5 Biotechnology Centre, University Eduardo Mondlane, Maputo, Mozambique; 6 Institut Sénégalais de Recherches Agricoles, Laboratoire National d'Elevage et de Recherches Vétérinaires, Service de Bio-Ecologie et Pathologies Parasitaires, Dakar-Hann, Sénégal; 7 Centre de Coopération Internationale en Recherche Agronomique pour le Développement (CIRAD), UMR Contrôle des Maladies Animales Exotiques et Emergentes (CMAEE), Montpellier, France; 8 L'Institut National De La Recherche Agronomique (INRA), UMR 1309 CMAEE, Montpellier, France; 9 Global Alliance for Veterinary Medicines (GALVmed), Doherty Building, Edinburgh, United Kingdom; 10 Ceva Santé Animal, Libourne, France; Federal University of São Paulo, Brazil

## Abstract

**Background:**

Diagnosis of African animal trypanosomosis is vital to controlling this severe disease which hampers development across 10 million km^2^ of Africa endemic to tsetse flies. Diagnosis at the point of treatment is currently dependent on parasite detection which is unreliable, and on clinical signs, which are common to several other prevalent bovine diseases.

**Methodology/Principle Findings:**

the repeat sequence of the GM6 antigen of *Trypanosoma vivax* (TvGM6), a flagellar-associated protein, was analysed from several isolates of *T. vivax* and found to be almost identical despite the fact that *T. vivax* is known to have high genetic variation. The TvGM6 repeat was recombinantly expressed in *E. coli* and purified. An indirect ELISA for bovine sera based on this antigen was developed. The TvGM6 indirect ELISA had a sensitivity of 91.4% (95% CI: 91.3 to 91.6) in the period following 10 days post experimental infection with *T. vivax*, which decreased ten-fold to 9.1% (95% CI: 7.3 to 10.9) one month post treatment. With field sera from cattle infected with *T. vivax* from two locations in East and West Africa, 91.5% (95% CI: 83.2 to 99.5) sensitivity and 91.3% (95% CI: 78.9 to 93.1) specificity was obtained for the TvGM6 ELISA using the whole trypanosome lysate ELISA as a reference. For heterologous *T. congolense* field infections, the TvGM6 ELISA had a sensitivity of 85.1% (95% CI: 76.8 to 94.4).

**Conclusion/Significance:**

this study is the first to analyse the GM6 antigen of *T. vivax* and the first to test the GM6 antigen on a large collection of sera from experimentally and naturally infected cattle. This study demonstrates that the TvGM6 is an excellent candidate antigen for the development of a point-of-treatment test for diagnosis of *T. vivax*, and to a lesser extent *T. congolense*, African animal trypanosomosis in cattle.

## Introduction

African animal trypanosomosis (AAT) is a devastating livestock disease costing approximately $1 -2 billion per annum in Africa [1]. AAT is caused by the tsetse-fly-transmitted (*Glossina* sp.) protozoan parasites *Trypanosoma congolense, T. vivax* and to a lesser extent, *T. brucei brucei* and by the non-tsetse transmitted trypanosomes such as *T. evansi*, the most widely distributed animal pathogenic trypanosome, which is the causative agent of surra [2]. Furthermore, *T. vivax*, in addition to being transmitted by tsetse flies, can also be transmitted by biting flies in South America and regions of Africa non-endemic to tsetse [3,4]. 

Disease progression is dependent on host factors as well as on parasite species and strain. The most prevalent clinical signs include anaemia, weight loss, reduced productivity, infertility and abortion [5]. However, symptoms are too varied and non-specific to be a reliable basis for diagnosis of AAT. 

Unlike human African trypanosomosis, where a lateral flow device prototype has recently been developed, no point-of-treatment test exists for AAT [6,7]. The current gold standard for diagnosis of AAT is examination of blood by light microscopy for the presence of parasites. The blood can be concentrated (usually by centrifugation) to improve sensitivity [8]. However, the usefulness of parasitological diagnosis is limited in chronic infections where the parasitaemia is low and intermittent. Even during acute infection, antigenic variation results in waves of parasitaemia which could easily be missed if sampling is only performed a single time [9]. 

In terms of serological diagnosis, the indirect fluorescent antibody test (IFAT) was one of the first antibody detections tests to be used for diagnosis of AAT. The IFAT is both sensitive and specific, but not species-specific [10]. Furthermore, the IFAT is not quantitative, requires fluorescent-enabled microscopes, and antigen preparation is not standardised. Antibody ELISA using whole trypanosomal lysate (WTL) was subsequently developed as a serological test for AAT [11]. Although it shows high sensitivity and specificity, the WTL ELISA is not species-specific and, again, standardisation of antigen production proves difficult. For these reasons, the WTL ELISA is not readily adaptable to an immune-chromatographic test format required for point of treatment diagnosis in field situations. However, partial purification of the crude lysate allows higher specificity [12]. Still, the general problem with antibody detection tests is that they do not only detect active infections, since antibodies against trypanosomes persist after treatment or self-cure. 

On the other hand, antigen detection ELISAs developed for AAT suffer from low sensitivity and low species-specificity as confirmed with experimental infections [13,14].

The GM6 antigen was originally identified in African trypanosomes by screening a *T*. *b. gambiense* cDNA library with infected bovine sera [15]. It has been shown that the GM6 antigen is an invariant antigen, associated with the flagellum and expressed in both procyclic and bloodstream forms (BSF) of the parasite [15]. The GM6 antigen contains a 68 amino acid repeat motif which is partially conserved in *T. brucei*, *T. congolense* and *T. vivax*. It has been noted that the GM6 antigens are part of the calpain superfamily, albeit with unusual repeat sequences and are unlikely to be active enzymes [16]. To date, the *T. vivax* GM6 and *T. congolense* GM6 antigens have not been tested in an ELISA to diagnose bovine trypanosomosis. Furthermore, previous studies of the GM6 antigen ELISA have been limited to small sets of infected sera, and almost exclusively from experimental infections. 

Currently, the treatments available for AAT are not species-specific. However, since no field diagnostic test is currently available for any animal trypanosome infections, diagnosis of *T. vivax* infection would be a good beginning. Primarily, the goal would be to incorporate antigens from both *T. congolense* and *T. vivax* into a pan-trypanosome field diagnostic test. Secondly, specific detection of *T. vivax* would be useful since this parasite is prevalent in both East and West Africa, in both tsetse endemic and non-endemic regions, as well as in South America. *T. vivax* is also responsible for haemorrhagic outbreaks of AAT, which would benefit from quick diagnosis [17]. Also, the natural habitat of tsetse is being reduced by climate change, encroaching human settlements and tsetse eradication programs [18]. For this reason, it is foreseeable that *T. vivax* could become more prevalent than *T. congolense* given that it does not require tsetse for transmission. Indeed, this has already been observed in the northern arid Djibo region of Burkina Faso [19]. 

For these reasons, in the current study, the repeat sequences of the GM6 proteins of *T. vivax* (TvGM6: TvY486_1101010) and *T. congolense* (TcoGM6: TcIL3000.11.1030) were recombinantly expressed, and purified. Sequencing of the TvGM6 genes from isolates from both East and West Africa showed high conservation despite the fact that *T. vivax* is known to be highly genetically diverse [20,21,22,23,24,25]. The purified GM6 antigens were subsequently used in an indirect ELISA that was optimised for detection of trypanosome infection in bovine sera. Sera from experimental infections using strains of *T. vivax* and *T. congolense* from both East and West Africa were tested in an indirect ELISA with the two GM6 antigens to determine the kinetics of infection. In addition, large collections of field sera were tested in order to determine the specificity and sensitivity of the TvGM6 indirect ELISA for both homologous and heterologous infections. 

## Materials and Methods

### Ethics statement

All mice procedures were carried out in strict accordance with the *French legislation* (Rural Code articles L 214-1 to L 214-122 and associated penal consequences) and European Union (Directive 2010/63/EU Protection of Animals Used for Scientific Purposes) guidelines for the care of laboratory animals and were approved by the Ethical Committee of Centre National de la Recherche Scientifique, Région Aquitaine and by the University of Bordeaux 2 animal care and use committee. All efforts were made to minimize animal suffering. 

For the cattle infections at ClinVet in South Africa, the study plan was submitted to the ClinVet Animal Ethics Committee (CAEC) and an approval certificate was issued authorizing the research facility to conduct the study. The study plan was designed to allow the use of the study animals in compliance with the ClinVet Policy on the ethical use of animals (CVI 08/03) using the South African National Standard “SANS 10386:2008 “The care and use of animals for scientific purposes” as a reference.

The protocol for cattle studies conducted by CIRDES (Centre International de Recherche-Développement sur l'Elevage en Zone subhumide, Bobo-Dioulasso, Burkina Faso) were reviewed and approved by the Scientific Committee of CIRDES, and complied with the requirements of ‘European Union Directive 2010/63/EU Protection of animals for scientific purposes; Requirements for establishments and for the care and accommodation of animals.

The research protocol for cattle infections at CB-UEM (Biotechnology Centre at the University Eduardo Mondlane, Maputo, Mozambique) was approved by the Scientific Board of the Veterinary Faculty of the Eduardo Mondlane University. The study was reviewed by The Mozambican Livestock National Directorate and handling of the animals and blood sampling were performed by approved staff, namely animal technicians and veterinary surgeons, according to the World Organization for Animal Health (OIE) guidelines for use of animals in research and education.

Additionally, the cattle studies conducted at CIRDES, ClinVet and CB-UEM were approved by the Scientific Committee of GALVmed (Global Alliance for Livestock Veterinary Medicine) in the frame of the Animal African Trypanosomosis Programme (*Aries code* 202040-101). 

### GM6 cloning, expression and purification

The *T. vivax* Y486 strain was initially isolated from a Zebu in West Africa (Nigeria) [26] and was kindly provided by the International Livestock Research Institute, Nairobi, Kenya. A fragment containing four copies of the repeat (270 bp) was amplified from the *T. vivax* GM6 gene (TvGM6: TvY486_1101010) using specific primers: Fwd: 5' GAA ATA CAG CAG CAA CAC GAT 3'; Rv: 5' GAA CTG CTC GTC CGC GTC AAG 3'. The amplicon was cloned into pGEX-4T-1 (GE Healthcare) in frame with the 5' GST-tag. A similar fragment (220 bp) of the *T. congolense* GM6 (TcoGM6: TcIL3000.11.1030) was synthesised commercially, due to cloning difficulties, (ProteoGenix, Oberhausbergen, France) and cloned into pGEX-4T-1. Recombinant vectors were used to transform *Escherichia coli* BL21 Star ™ (DE3) (Invitrogen, Saint-Aubin, France) for expression. Cultures in mid-exponential growth phase were induced with 0.4 mM IPTG for 3-4 hrs. Recombinant fusion protein was present in the supernatant of cell lysate. Cells were lysed with an extraction buffer (50 mM Tris-Cl, pH 8.5, 100 mM NaCl, 1 mM EDTA) supernatants bound to Glutathione Sepharose 4B (GE Healthcare) for 1 hr at room temperature (RT) with gentle agitation. The resin was washed five times in extraction buffer (10 column volumes) and resuspended in 1 ml thrombin cleavage buffer (50 mM Tris pH 8.0, 150 mM NaCl, 2.5 mM CaCl_2_). Thrombin (10 units, Sigma) was added to the resin and incubated overnight at RT with gentle agitation. Fractions containing cleaved GM6 protein were collected, and concentration estimated by Bradford protein assay [27]. 

### Trypanosome strains and serum origins

Sera were obtained from several sources. Sera from *T. congolense* experimental infections with the strain MozO2J (isolated in Mozambique; L. Neves, 2012) and KONT2/133 (isolated in Cameroon; [28]) were obtained from novel trypanocide efficacy studies conducted at ClinVet (Bloemfontein, South Africa) by GALVmed (Global Alliance for Livestock Veterinary Medicine). For these studies, animals were treated with either 7 mg/kg diminazene diaceturate or 1 mg/kg isometamidium chloride and novel compounds under evaluation for efficacy against *T. congolense and T. vivax*. *T. vivax* infections were conducted at CIRDES (Centre International de Recherche-Développement sur l'Elevage en Zone subhumide, Bobo-Dioulasso, Burkina Faso) by GALVmed, using strains isolated in West Africa (Komborodougou and Napie, isolated in Ivory Coast by S. Yao Loukou; Gando Bongaly, isolated in Togo by S. Boma) provided by Z. Bengaly. Animals were treated with 3.5 mg/kg of diminazene diaceturate. *T. vivax* experimental infection sera were also obtained from infections conducted at ILRI (Nairobi, Kenya) using the strains IL2172 and IL3769 (Ugandan origin [26]) and were provided by a co-author and E. Authié. Additional *T. vivax* experimental infections were conducted in Mozambique at CB-UEM using a local isolate (175J) and the Y486 reference strain [29]. Corresponding parasitaemia was estimated by phase contrast buffy coat [30]. *T. vivax*-infected field sera from Western Senegal, characterised by whole trypanosome lysate ELISA were provided by co-authors [31]. *T. vivax*-infected field sera from Ethiopia characterised by ITS-PCR were provided by co-authors [3]. *T. congolense*-positive field sera (buffy coat and 18s PCR) and negative sera from animals in a tsetse-free region were collected in the South of Mozambique (Biotechnology Centre, University Eduardo Mondlane, Maputo, Mozambique). 

### ELISA

Indirect ELISA was optimised for type and concentration of blocking agent, coating antigen concentration and secondary antibody concentration. Recombinant GM6 antigen was purified as described above. *T. congolense* (IL3000) and *T. brucei brucei* (AnTat 1) BSF parasites were obtained from *in vitro* culture [32,33]. *T. vivax* (Y486) BSF parasites were propagated in mice and purified either by centrifugation [34] or DE-52 ion-exchange chromatography [35], from which whole parasite lysate was prepared by osmotic lysis. Briefly, antigen (4 µg/ml for TvGM6, 10 µg/ml for TcoGM6 and whole trypanosome lysate) was diluted in carbonate coating buffer (50 mM carbonate buffer, pH 9.6) and plates coated with 100 µl per well and incubated overnight at 4°C. Blocking buffer (1% horse serum in PBS) was added to the wells (200 µl/well) and the plate incubated at 37°C for 1 h. Primary sera diluted in blocking buffer (1/100) were added to the wells in duplicate (100 µl/well) and incubated at 37°C for 2 h. The plates were washed with 0.05% PBS-Tween-20 using either a squeeze bottle or an automated microplate washer (ThermoFisher Scientific WellWash 4 Mk 2 MicroPlate Washer). Secondary antibody, rabbit anti-bovine horse-radish peroxidase conjugate (Sigma) diluted in blocking buffer (1/4000), was added to the wells (100 µl/well). Plates were washed as before and commercial ABTS substrate-chromogen solution (KPL) added (100 µl/well). Optical density (OD 405 nm) was measured approximately 10-15 min after addition of the substrate (FLUOStar OPTIMA fluorescence plate reader). Readings were considered acceptable when the OD values for the positive and negative control samples fell within specific ranges, with a coefficient of variance less than 10%.

Known strong positive and negative bovine serum samples (based on previous ELISAs) were added to each plate allowing calculation of the percent positivity (PP) for each sample [36]. For experimental infection sera, combined weighted estimates of sensitivity from sequential sera of nine infected animals and the weighted standard errors with 95% confidence limits were calculated according to Eisler et al., [13]. The significance of the differences observed between the TvGM6 and TvWTL ELISA was compared using the McNemar test using GraphPad Software (GraphPad Software Inc.). For field sera, cut-offs were established using a minimum of 10 PCR negative bovine sera from areas non-endemic to tsetse (peri-urban Maputo, Mozambique). Cut-offs were calculated as the mean PP added to two standard deviations. Sera were tested in duplicate, and each experiment was performed at least twice, allowing estimation of the standard error for positive and negative samples from each region. 

### Immunofluorescence

Anti-TvGM6 and anti-TcoGM6 sera were obtained by immunising mice at two week intervals with initially 50 µg of purified recombinant protein (in Freund’s Complete adjuvant), followed by two boosters of 25 µg (Freund’s Incomplete adjuvant). Parasite pellets were washed in phosphate saline with glucose (PSG), resuspended in 320 µl fixing solution (3% formaldehyde in PBS, freshly prepared) and incubated at RT for 10 min. Fixing was blocked using 1 M glycine-Cl (80 µl) and incubation at RT for 10 min. Fixed parasite suspension (20 µl) was added to slide wells. Dried slides were blocked using 0.5% BSA-PBS (50 µl/well) at RT for 10 min, followed by permeabilisation of the cells using 0.1% Triton X-100 in PBS (20 µl) for the same time. Primary antibody diluted in blocking buffer (20 µl) was added to each well and incubated in a humidified atmosphere for 1 hr. Primary antibodies used were either mouse anti-TvGM6 sera (1/2000) or mouse anti-TcoGM6 sera (1/2000), and rabbit anti-paraflagellar rod (1/50). Slide wells were washed using blocking solution (3x50 µl). Secondary antibody diluted 1/100 in blocking buffer (20 µl) was added to slide wells and incubated for 60 min. Secondary antibodies used were Alexa Fluo 488 conjugated goat anti-mouse IgG and Texas Red® conjugated goat anti-mouse IgG (Invitrogen, Carlsbad, CA, USA). DAPI (20 µl/ slide well) diluted in PBS (final 0.5 µg/ml) was added to each slide well. Slides were viewed using a Zeiss Axio Imager Z1 fluorescent microscope and images captured using the MetaMorph® software (Molecular Devices, CA, USA) at a total magnification of 100x. 

## Results

### TvGM6 is conserved within *T. vivax* isolates and is possibly flagellar-associated

**Figure 1:**
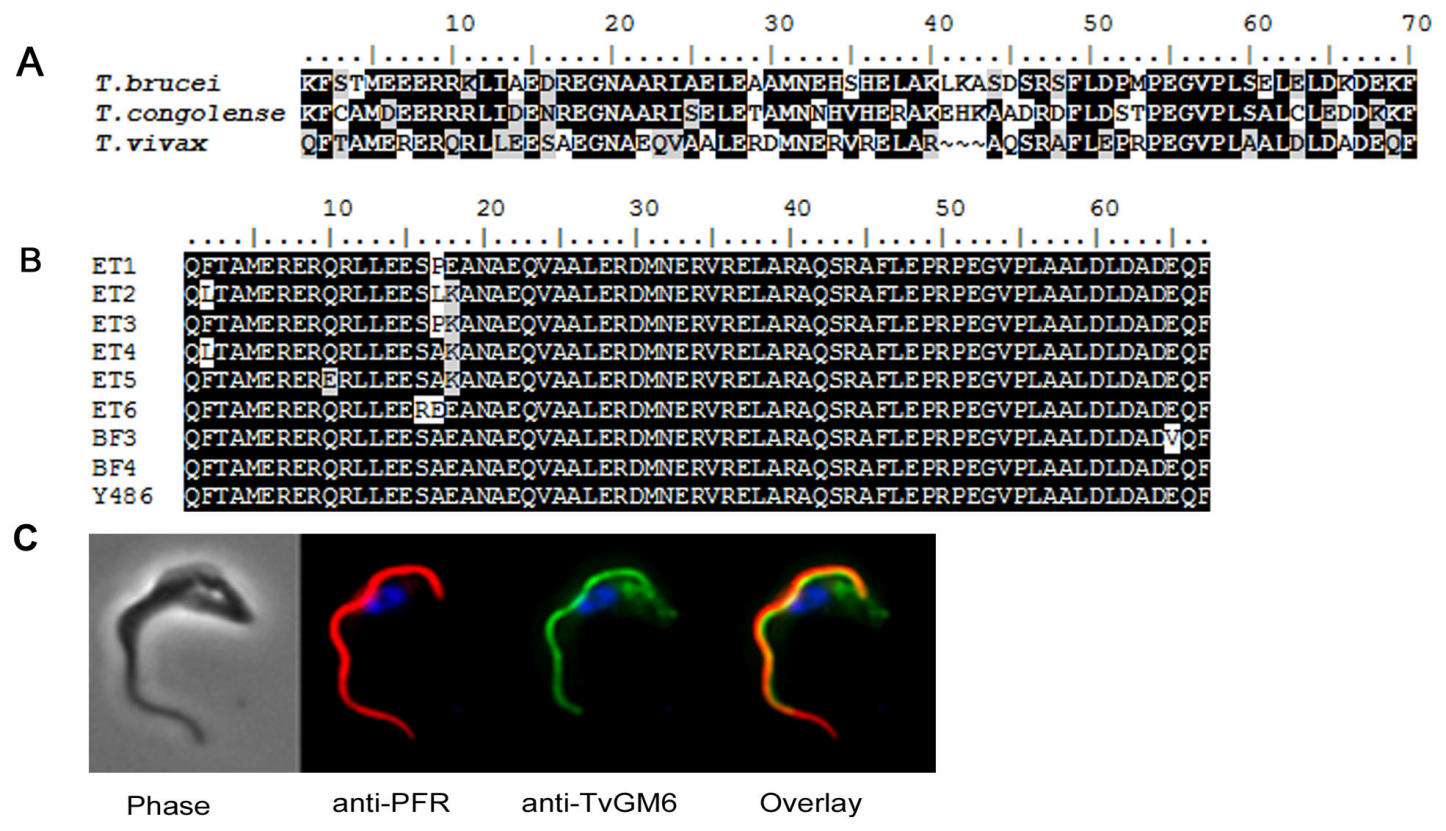
TvGM6 is a homolog of the protein found in other trypanosomes, is conserved within *T*, *vivax* isolates and is flagellar associated. (A) Alignment of the GM6 repeat motif from *T. brucei brucei* (427), *T. congolense* (IL3000) and *T. vivax* (Y486). (B) Sequence alignment of a single repeat of the TvGM6 sequence from *T. vivax* isolates originating from Burkina Faso (BF) and Ethiopia (ET). The *T. vivax* Y486 reference strain is shown. Background colour indicates conservation (black), similarity (grey) and differences (white) in amino acid sequence. (C) Immunofluorescence microscopy of *T. vivax* Y486 bloodstream forms showing partial co-localisation of anti-TvGM6 antibodies (green) with anti-paraflagellar rod (PFR) antibodies (red).

TvGM6 is a homolog of the genes found in *T. brucei brucei* (Tb11.57.0008) and *T. congolense* (TcIL3000.11.1030). However, the TvGM6 repeat sequence only shares 51 and 55% identity and 72 and 64% similarity with the homologs of *T*. *b. brucei* and *T. congolense*, respectively (Figure 1A). Furthermore, the number of repeats of the 68 amino acid motif differs between the different species. The TvGM6 has 11 copies of the repeat compared to 60 in *T*. *b. brucei* and 9 in *T. congolense*. In order to determine the level of variability in the TvGM6 gene within the *T. vivax* species, the repeat was sequenced from several *T. vivax* strains isolated in different regions. As is evident from Figure 1B there were, at most, two amino acid substitutions in a single copy of the TvGM6 repeat. *T. vivax* strains from Burkina Faso had only one amino acid substitutions in comparison to the *T. vivax* Y486 reference strain. 

Immuno-localisation studies (Figure 1C) showed that anti-TvGM6 antibodies partially co-localised with anti-paraflagellar rod antibodies. This indicated that the TvGM6, like the homologs in other trypanosome species, is likely to be associated with the flagellum. The co-localisation with the anti-PFR antibodies was only partial, indicating that the localisation of the TvGM6 is not entirely flagellar, and it is possible that the TvGM6 may be present in the flagellar attachment zone. TvGM6 was detected in both bloodstream form and procyclic parasites (data not shown). An idential localisation pattern was observed using anti-TcoGM6 antibodies (data not shown). 

### TvGM6 and TcoGM6: Detection of infection during *T. vivax* and *T. congolense* experimental infections

**Figure 2:**
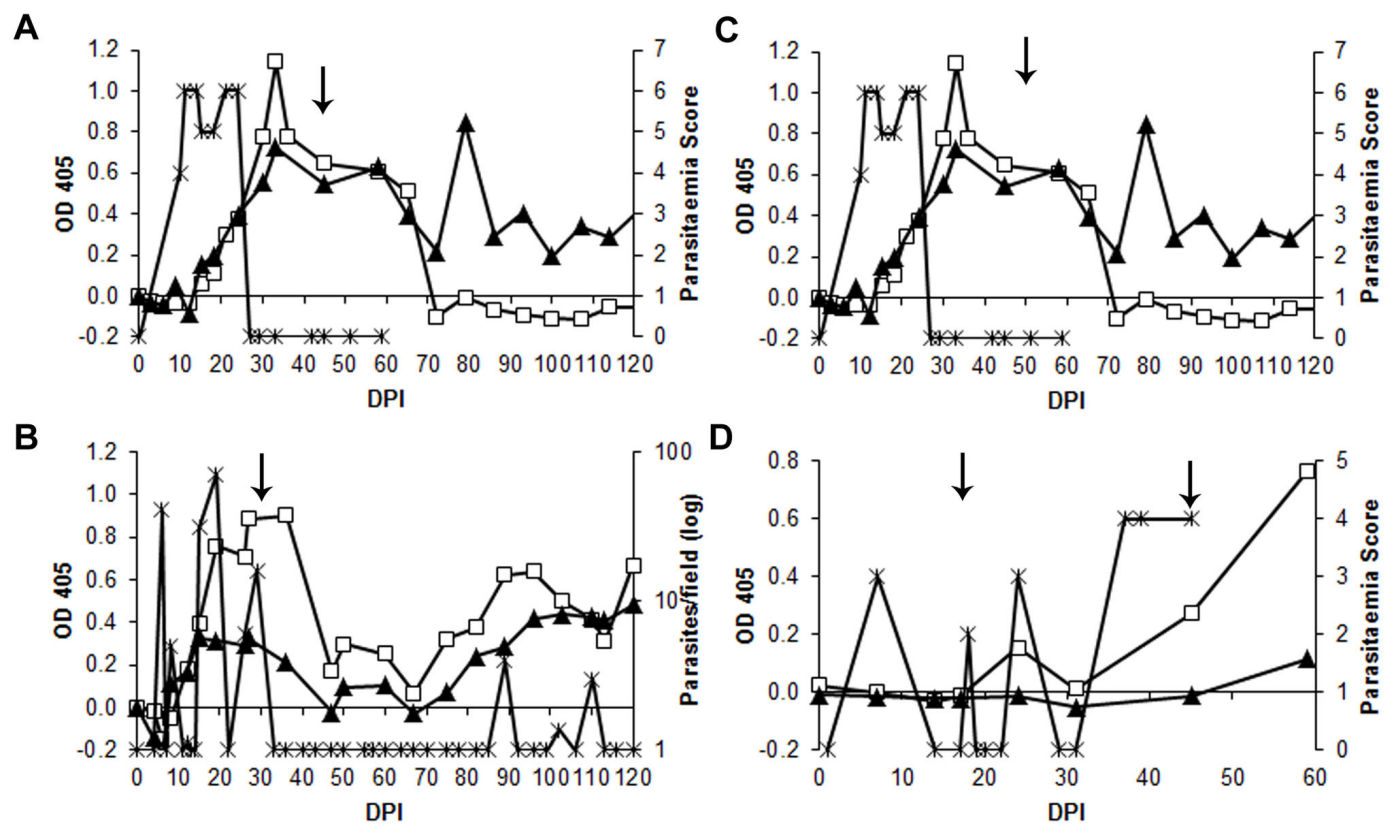
Representative TvGM6 and TcoGM6 ELISA analysis of longitudinal experimental infection sera with (A, B) *T. vivax* and (C,D) *T. congolense* in individual animals. TvGM6 ELISA using sera from infections with (A) *T. vivax* IL2172 (drug-sensitive) and (B) *T. vivax* Komborodougou (drug-resistant). TcoGM6 ELISA using sera from infections with (C) *T. congolense* O2J (drug-sensitive) and (D) *T. congolense* KONT2/133 (drug-resistant). All animals were treated with 3.5mg/kg of diminazene diaceturate at the dates indicated by the arrows, (D) was treated a second time with 1 mg/kg isometamidium chloride. For figures (A), (C) and (D) parasitaemia score can be related to approximate amounts of parasites as follows: 2 (1-10/preparation), 3(1-2/field), 4 (1-10/field), 5 (10-50/>50 field), 6 (>100/field). TvGM6 or TcoGM6 ELISA OD (£), whole trypanosome lysate ELISA OD () and parasitaemia () are indicated. Arrows indicate trypanocidal treatment, and the x-axis is the threshold for positivity.

In total, sera from nine *T. vivax* experimental infections were tested with the TvGM6 ELISA: three with West African isolates, two with East African isolates from Uganda, two with a Mozambican isolate and two with the Y486 reference strain (Nigerian origin [29]). A representative result (only one animal per infection) of the TvGM6 indirect ELISA with a *T. vivax* experimental infection compared to the ELISA using whole trypanosome lysate of *T. vivax* (TvWTL) is shown in Figure 2A and 2B. 

The TvGM6 ELISA was positive 20 to 30 days post infection (DPI), and reduced to baseline approximately 20 to 30 days post-treatment when the animal was successfully treated (Figure 2A). In Figure 2B, the TvGM6 ELISA was tested with samples from an infection using a drug-resistant strain. In this case, the parasitaemia decreased post-treatment and no parasites were detected between 35 and 90 DPI. However, a relapse was observed around 91 DPI, when parasites were again detected in the blood. The antibody response against TvGM6 decreased at 70 DPI (approximately one month after treatment) and increased again 80 DPI, 15 days prior to the detection of parasites by microscopy. These results suggest that this antigen could be used to diagnose an active infection, and, furthermore, would be a good indicator of the efficacy of treatment. The TvWTL ELISA did not decrease as quickly (or at all) as the TvGM6 ELISA after treatment. 

Indirect ELISA using TcoGM6 during *T. congolense* experimental infection is shown in Figures 2C (drug-sensitive strain) and 2D (drug-resistant strain). A peak of antibody response was observed at 25 DPI, and decreased dramatically post-treatment (Figure 2C), however, this was only observed for sera from five out of a total of eleven experimental infections. In the other six experimental infections, no ELISA response to TcoGM6 was observed (data not shown). Therefore, detection of early infection using TcoGM6 ELISA, during the first waves of parasitaemia, was inconsistent. As can be seen in Figure 2D, the initial antibody response is barely detectable 25 DPI, whereas the response continues to increase after several waves of parasitaemia. 

Comparative values for positivity of the TvGM6 ELISA, whole trypanosome lysate (TvWTL) ELISA, and buffy coat during experimental infection with *T. vivax* are shown in Table 1. The TvWTL ELISA showed approximately double the sensitivity of either the TvGM6 ELISA or the buffy coat method very early in infection. However, the two-tailed P-value calculated using the McNemar test was 0.2482 indicating that the difference between the TvWTL and TvGM6 ELISAs was not significant. Later than 10 DPI, the TvGM6 ELISA and the TvWTL ELISA were comparable at approximately 90% of sensitivity (no significant difference P-value = 0.6831) while the buffy coat method was only 24% sensitive. Finally, the TvGM6 ELISA results were 4.5 times less likely to be positive 30 days post-treatment than the TvWTL ELISA results. The McNemar test P-value was 0.0133 indicating that the TvGM6 ELISA was significantly less likely to detect false positives post-treatment than the TvWTL ELISA. 

**Table 1:** Sensitivity of TvGM6 ELISA compared to TvWTL ELISA and buffy coat in *T. vivax* experimental infections.

**Point of Infection**	**Test**	**No. of cattle**	**No. of Samples**	**Sensitivity ^a^ %**	**95% CI** ^b^
**< 10 DPI ^d^**	TvGM6 ELISA	9	26	24.0	17.5 to 30.4
	TvWTL^c^ ELISA	9	26	34.6	29.8 to 39.4
	Buffy Coat	9	32	15.6	14.7 to 16.6
**> 10 DPI ^d^**	TvGM6 ELISA	9	116	91.4	91.3 to 91.6
	TvWTL ELISA	9	116	90.5	90.4 to 90.7
	Buffy Coat	9	213	23.9	23.8 to 24.0
**30 DPT ^e^**	TvGM6 ELISA	4	21	9.1	7.3 to 10.9
	TvWTL ELISA	4	21	42.9	9.9 to 75.5
	Buffy Coat	2	15	0	0

^a^Combined sensitivity values were weighted for the number of samples per animal

^b^95% confidence interval

^c^ *T. vivax* whole trypanosome lysate

^d^days post infection

^e^days post treatment

### TvGM6: High specificity and sensitivity in field infections

TvGM6 indirect ELISA was used to test sera from cattle infected with *T. vivax* originating from Ethiopia, Senegal and Mozambique (Table 2). TvGM6 ELISA showed a mean (weighted) sensitivity of 91.5% (95% CI: 83.2 to 99.5) and a mean (weighted) specificity of 91.3% (95% CI: 78.9 to 93.1) in comparison to the TvWTL ELISA. In terms of a comparison to PCR (not shown), for the Ethiopian field sera, the TvGM6 ELISA had a sensitivity of 79% compared to PCR positive samples (268 tested), whereas the WTL ELISA had a lower sensitivity of 68%. The lower specificity for the Senegalese and Ethiopian sera (85.4 ± 6.9%) could be attributed to the fact that the negative sera were obtained from an endemic region, i.e. it cannot be excluded that animals were previously infected and treated. Since the TvGM6 indirect ELISA requires a minimum of 20-30 days post treatment to return to baseline values (as seen from the experimental infections), it is possible that some animals which test serologically positive had been infected and treated within one month of serum collection. 

**Table 2:** Sensitivity and specificity of TvGM6 ELISA compared to the whole trypanosome lysate ELISA in *T. vivax* field infections.

	**Region**	**Samples**	**Sensitivity (%**)** ± SD** ^a^	**Mean** (**95% CI**) ^b^
**Positives**	Ethiopia	179	94.4 ± 5.5	91.5 (83.2 to 99.5)
	Senegal	211	89.1 ± 7.4	
	**Region**	**Samples**	**Specificity (%**)** ± SD** ^a^	**Mean** (**95% CI**) ^b^
**Negatives**	Mozambique	84	96.4 ± 9.0	91.3 (78.9 to 93.1)
	Ethiopia	36	86.1 ± 2.0	
	Senegal	41	85.4 ± 6.9	

^a^standard deviation

^b^95% confidence interval

In addition, the TvGM6 ELISA was negative (below the cut off for positivity) when tested with bovine sera from animals infected with *Anaplasma marginale, Babesia bigemina* and *Theileria buffeli* (Marula) (results not shown)*.*

### TvGM6 : Cross-reactions with heterologous *T. congolense* infection

The TvGM6 ELISA was also tested with sera from *T. congolense* experimental infections, and produced a pattern similar to the TcoGM6 indirect ELISA (data not shown), with a peak of antibody response 25 DPI and a rapid decrease below the threshold post-treatment. However, the TvGM6 ELISA was consistently weaker than the TcoGM6 ELISA when detecting heterologous infection. Due to this initial finding of a cross-reaction, it was decided to test the TvGM6 indirect ELISA with *T. congolense*-infected field sera to determine the sensitivity of this test for the heterologous infection and the results are shown in Table 3. The sensitivity of TvGM6 ELISA with *T. congolense* field infections gave a mean sensitivity of 85% (95% CI: 76.8 to 94.4) in comparison to the whole trypanosome lysate ELISA. 

**Table 3:** Sensitivity of TvGM6 ELISA against *T. congolense* field sera from two different regions.

**Region**	**Total**	**Sensitivity (%**)** ± SD** ^a^	**Mean** (**95% CI**) ^b^
Ethiopia	28	89.6 ± 4.7	85.1 (76.8 to 94.4)
Mozambique	165	81.7 ± 2.7	

a standard deviation

b 95% confidence interval

## Discussion

Diagnosis of AAT is currently made on the basis of clinical signs, which are common to several other bovine pathogens, resulting in frequent misdiagnosis. Currently, no point-of-treatment diagnostic tool exists for diagnosis for either *T. congolense* or *T. vivax* infections. Furthermore, detection of either parasite would require the same intervention since there is no difference in treatment. In the current study, the immunodiagnostic potential of the *T. vivax* GM6 antigen for the detection of *T. vivax* has been explored as the first step towards a pan-trypanosome point-of –treatment diagnostic tool.

In the current study, the repeat motif of the GM6 antigens of *T. vivax* (TvGM6) and *T. congolense* (TcoGM6) were expressed and purified and their immunodiagnostic potential tested in an indirect ELISA with sera from cattle infected with either *T. vivax* or *T. congolense*. *T. vivax* is known to be quite genetically diverse and several studies have shown that West African and South American *T. vivax* strains are genetically distinct from East African isolates [21,22,23,37]. For this reason, sequencing of the TvGM6 gene from isolates originating from both East and West Africa was deemed necessary to confirm that the sequence was sufficiently conserved to allow detection of infected animals in both regions. 

As shown with the *T. brucei* GM6, this study confirmed that the TvGM6 was present in both bloodstream form and procyclic parasites. Immuno-localisation of the TvGM6 indicated that, similar to the TbGM6, the *T. vivax* antigen was likely to be located in the flagellar attachment zone. 

Previous preliminary diagnostic studies had been done with the GM6 antigen from different trypanosome species, including a recombinant beta-galactosidase-*T*. *b. gambiense* GM6 fusion protein which showed high immunodiagnostic sensitivity with sera from *T. brucei* and *T. congolense*-infected cattle [15]. The *T*. *b. brucei* GM6 (TbbGM6) antigen was tested in an antibody ELISA for *T. evansi* infection, but was not sufficiently sensitive [38]. However, it was useful in a competitive ELISA using *T. evansi* infected bovine or buffalo sera, but not wallaby, pig, dog or horse-infected sera [38]. Thuy et al., (2011) searched for repeat antigens of *T. congolense* for use in a diagnostic test for *T. evansi*. They identified TcoGM6 and TbbGM6 as potential antigens since both showed higher reactivity to *T. evansi* –infected water buffalo sera than other repeat antigens [39].

It is known that repeat antigens are good targets for B-cell responses [40]. This may explain why the TvGM6, which is a minor, insoluble antigen, has a high sensitivity in an indirect ELISA. In fact, antibody responses against repeat proteins of several protozoan parasites have been found, including for malaria [41], Chagas disease [42] and leishmaniasis [43]. 

In the current study, sera from longitudinally-followed experimental infections allowed definition of the kinetics of the antibody response to these antigens, including the length of the period post-infection before antibodies became detectable (pre-patent period) and, most importantly, the time necessary for the antibody response to decrease below the threshold post-treatment. Sera obtained from naturally infected animals in the field were tested in order to determine the level of sensitivity and specificity of the GM6 antigen indirect ELISA. To ensure that the GM6 ELISA was not strain or isolate specific, sera from distinct geographic regions were tested. This is especially significant in the case of *T. vivax*, since the TvGM6 ELISA gave similar results for experimental infections conducted with strains originating from Burkina Faso, Nigeria, Uganda and Mozambique. 

Based on the experimental infections, it is clear that the antibody response against TvGM6 decreases to baseline approximately one month after treatment. This could imply that certain field sera which tested negative on PCR were positive on the GM6 indirect ELISA due to persistence of antibodies after treatment. Previous studies have shown that antibodies against the WTL can persist for 10-13 months post treatment [44,45]. However, Authié et al. (1993) indicated that although animals treated 10 months previously tested positive in a WTL ELISA, a western blot with the WTL indicated that only antibodies recognising a few specific antigens were still present. Given that the GM6 is a relatively minor, insoluble antigen, it is probable that a certain level of parasitaemia is necessary to stimulate a B-cell response. In the absence of this stimulation, when the parasitaemia drops beneath the necessary parasite load, the antibody response is short-lived. 

Experimental infections showed that the antibody response to the TvGM6 was detected at the earliest, 10 days post infection, during which period the PCR results were likely to be positive. Therefore, the TvGM6 indirect ELISA may show false negatives for animals which have been recently (less than 10 days) infected. The onset of anaemia, the most prevalent clinical sign, occurs at approximately at the same time as the emergence of detectable parasitaemia (1-3 weeks depending on infecting strain, infective dose and host genetics) [5]. Therefore, the detection of infection using the TvGM6 ELISA would be similar to the clinical pre-patent period. It was found that the TvGM6 ELISA has a similar sensitivity to the WTL ELISA later than 10 days post infection, but the antibody response to TvGM6 decreased less than one month post treatment, whereas the antibodies against the WTL tended to persist for a longer period. 

The case of the TvGM6 antigen cross-reacting with *T. congolense*-infected sera is probably due to the few regions which are sufficiently conserved to provide common epitopes between the two species. However, the cross-reaction detected is lower than with the homologous antigen. Furthermore, this cross-reaction of the TvGM6 ELISA with *T. congolense*-infected sera indicates that the TvGM6 ELISA alone cannot be used for species-specific diagnosis. However, since the GM6 ELISA was consistently stronger when the homologous antigen was used, testing sera with both the TcoGM6 ELISA and TvGM6 ELISA would allow a relative response to be measured and, therefore, tentative diagnosis of the trypanosome species.

TcoGM6 gave inconsistent results when tested with sera from experimental *T. congolense* infections, and subsequently did not always detect early infection. However, the antibody response did increase following several waves of parasitaemia which may imply that the TcoGM6 would only detect secondary or re-current infections. Although the mechanism responsible is unknown, this phenomenon has already been encountered with the HSP70-based indirect ELISA and inhibition ELISA [46,47]. 

In summary, the TvGM6 ELISA allowed reliable detection of antibody response around 10-20 DPI with *T. vivax* infections, and decreased below the threshold less than one month following treatment, to date, the best characteristic of a trypanosome indirect ELISA. These results indicate the TvGM6 ELISA would essentially allow detection of active *T. vivax* infection, making it ideal for a point-of-treatment diagnostic tool. This would be of particular significance in South America, where a high proportion of the cattle are infected but display no symptoms [48]. Given that the TvGM6 ELISA does not detect infections one month post failed treatment prior to relapse (when the parasite is not detectable in the blood), it is likely that the test would not be positive for the apparent silent infections in South America and this would allow the discrimination of only active infections requiring treatment. However, due to the fairly rapid antibody decay post treatment, the TvGM6 ELISA would not be suitable as a test for exposure to *T. vivax*. Furthermore, the TvGM6 ELISA detected a failure of treatment at least 15 days prior to the buffy coat method. Finally, the TvGM6 indirect ELISA showed high sensitivity and specificity values in field infections for *T. vivax* infections and slightly lower values for *T. congolense* infection. 

For *T. congolense*, it was noted that the TvGM6 ELISA worked better in field conditions than experimental infections, possibly due to the fact that only secondary or recurrent infections are detected using this test. This hypothesis still needs to be confirmed. As mentioned previously, the ultimate goal would be to incorporate antigens from both *T. congolense* and *T. vivax* into a pan-trypanosome point-of-treatment diagnostic tool. To this end, the data presented in this study motivate that the TvGM6 would be a good antigen for the detection of *T. vivax* infections, but not necessarily for *T. congolense* infections. For detection of *T. evansi*, it was shown that the *T. brucei* GM6 did not give sufficiently high specificity (approximately one-third) [38], therefore, it is unlikely that the *T. vivax* or *T. congolense* GM6 antigens would perform better. A difficulty with the field sera was first to determine which method to use as a reference since the different sera collections had been characterised using different techniques (either ITS-PCR, 18s PCR, whole trypanosome lysate ELISA, or buffy coat). Given that these methods are not infallible, comparative sensitivity and specificity values of the TvGm6 ELISA should be interpreted carefully. It is possible that some field infections detected as false negatives with the TvGM6 ELISA were at a very early point in infection, since the experimental infections indicated that TvGM6 ELISA showed a very low sensitivity less than 10 days post infection. Conversely, a possible explanation for some false positives with the field sera is that some animals could have been treated less than one month prior to serum collection. Based on the TvGM6 ELISA in experimental infections, these animals would continue to test positive for one month post treatment even if treatment was successful. Although sera from *T. vivax* infections across East and West Africa were analysed during the course of this study, no analysis was done of South American *T. vivax* infections. This analysis would be useful *T. vivax* infection is estimated to be the third most economically important bovine parasitic infection in South America [4]. 

This study represents the first analysis of the GM6 antigen of *T. vivax* as a diagnostic candidate for AAT, and is the first to test the GM6 antigen with a wide range of both experimental and field *T. vivax*- infected sera from various locations. The data reported here demonstrate the potential of the TvGM6 antigen for the development of a point-of-treatment test for diagnosis of *T. vivax* in cattle. The TvGM6 ELISA could also be used for detection of *T. congolense*, albeit with lower sensitivity. 
